# Intrauterine Growth-Restricted Pig-Associated Testicular Transcriptome Analysis Reveals microRNA-mRNA Regulatory Networks

**DOI:** 10.3390/ani15172486

**Published:** 2025-08-24

**Authors:** Jiaxin Li, Kai Wang, Jianfeng Ma, Lijun Sun, Lili Niu, Ye Zhao, Lei Chen, Lixin Zhou, Jia Xue, Xiaofeng Zhou, Yan Wang, Linyuan Shen, Li Zhu, Mailin Gan

**Affiliations:** 1 Farm Animal Genetic Resources Exploration and Innovation Key Laboratory of Sichuan Province, Sichuan Agricultural University, Chengdu 611130, China; 2 State Key Laboratory of Swine and Poultry Breeding Industry, Sichuan Agricultural University, Chengdu 611130, China; 3 Key Laboratory of Livestock and Poultry Multi-Omics, Ministry of Agriculture and Rural Affairs, College of Animal and Technology, Sichuan Agricultural University, Chengdu 611130, China; 4 Chengdu Animal Disease Prevention and Control Center, Chengdu 610065, China

**Keywords:** intrauterine growth restriction, pig model, microRNAs, spermatogenesis disorder

## Abstract

Intrauterine growth restriction (IUGR) severely hinders the development of the livestock industry and impacts economic efficiency. MicroRNAs (miRNAs) participate in the epigenetic regulation of animal growth and development. Using IUGR pigs as a model, this study analyzed transcriptomic data from IUGR piglets to investigate the miRNA-mRNA regulatory network in their testes. Compared with NBW pigs, IUGR pigs exhibited reduced testicular volume, decreased weight, and abnormal testicular development. A total of 4945 differentially expressed mRNAs and 53 differentially expressed miRNAs were identified in IUGR testicular tissues, including 1748 downregulated and 3197 upregulated mRNAs, as well as 41 upregulated and 12 downregulated miRNAs. The integrated analysis of differentially expressed genes, miRNA target genes, and the miRNA-mRNA network revealed that IUGR may impair testicular development by disrupting cell cycle progression and apoptotic pathways, thereby hindering normal testicular cell growth. Furthermore, analysis of the miRNA-mRNA network indicated that miRNAs such as ssc-miR-23a, ssc-miR-29c, ssc-miR-193a-3p, and ssc-miR-574-3p could serve as potential marker miRNAs for IUGR testes, while YWHAZ, YWHAB, and PPP2CA may function as core target genes within this regulatory network. In conclusion, this study enhances our understanding of male reproduction in IUGR pigs and provides a theoretical foundation for preventing and treating IUGR-induced male reproductive disorders.

## Simple Summary

Intrauterine growth restriction (IUGR) is one of the major factors affecting the development of the animal husbandry industry, and an increased proportion of IUGR pigs impairs boar reproductive capacity. This study used IUGR piglets as a model, analyzed testicular transcriptomic data, and explored the IUGR-related miRNA-mRNA regulatory network. The results showed that IUGR led to reduced testicular volume and weight, as well as abnormal development, and identified 4945 differentially expressed mRNAs and 53 differentially expressed miRNAs. Analysis indicated that IUGR may interfere with testicular cell growth by affecting the cell cycle and apoptotic pathways, leading to developmental abnormalities. The study also identified potential signature miRNAs for IUGR and core target genes affecting normal cellular development. This research deepens the understanding of mechanisms by which IUGR affects porcine male reproduction and provides a theoretical foundation for preventing and treating related reproductive diseases.

## 1. Introduction

Intrauterine growth restriction (IUGR) refers to the phenomenon where the fetus grows at a slower rate, experiences impaired growth and development during pregnancy, and is born with a lower birth weight [1,2]. In pig production, birth weight is commonly used as a criterion to identify IUGR. IUGR is defined as a birth weight below the 10th percentile of littermates or an absolute weight less than 1.1 kg, accompanied by characteristic morphological abnormalities such as a “dolphin-like” forehead, protruding eyes, and a lean body profile [3]. In pig populations, IUGR predominantly presents as the asymmetric type (accounting for approximately 85%), which is characterized by fetal nutrient deficiency during late gestation. This condition leads to a brain-sparing effect, in which brain development is prioritized at the expense of the normal growth of organs such as the liver and muscles [4]. Current research indicates that IUGR piglets account for approximately 15–20% of newborn piglets [2], and the growth of IUGR piglets is accompanied by issues such as poor organ development [5], immune dysfunction [6,7], intestinal dysfunction [8,9], metabolic abnormalities [10,11], and impaired fertility [12,13], which not only affect the effectiveness of genetic breeding but also impact the economic efficiency of pig production.

In pigs, individuals with IUGR are often accompanied by impaired muscle development and delayed sexual maturation, ultimately leading to prolonged fattening periods, reduced feed conversion efficiency, and decreased meat quality [14]. Good reproductive performance is crucial for genetic breeding and also a prerequisite for ensuring the economic benefits of commercial pig production. It has been reported that intrauterine growth restriction can lead to gonadal dysplasia and sex hormone imbalance [13]. Current studies have shown that male piglets with intrauterine growth restriction (IUGR) exhibit impaired reproductive function during subsequent growth, characterized by reduced numbers of germ cells and semen volume, as well as decreased sperm quality and spermatogenesis efficiency [12,15]. The primary functions of the testes are to maintain spermatogenesis and hormonal balance [16], and normal testicular development is a prerequisite for sustaining male reproductive function. Abnormal testicular development leads to reduced reproductive capacity in male individuals, thereby affecting reproductive performance [17]. Current studies indicate that decreased testicular volume in IUGR piglets at birth results in impaired spermatogenic function in adulthood, compromising individual reproductive performance [18]. These adverse outcomes not only increase production costs but also reduce carcass value, thereby significantly weakening the economic efficiency of pig farming. Therefore, investigating the differences in testicular development between IUGR and normal piglets is critical for mitigating reproductive impairments in male IUGR pigs and offers a theoretical foundation for enhancing the economic efficiency of swine production.

MicroRNA (miRNA) serves as one of the mechanisms of epigenetic regulation and a key player in animal growth and development as well as gene expression regulation [19]. miRNAs are endogenous 21–23 nt small non-coding RNAs that primarily regulate gene expression at the post-transcriptional level [20]. Current studies have shown that the prenatal skeletal muscle-related miRNA-mRNA regulatory network in intrauterine growth-restricted (IUGR) pigs can serve as an indicator of prenatal fetal growth and postnatal carcass quality [21]. Differentially expressed miRNAs in the placenta and/or maternal circulation during preeclampsia (PE) and IUGR may act as biomarkers for predicting or diagnosing pregnancy complications [22]. Research indicates that IUGR piglets exhibit significant differences in testicular DMRT1 and SPP1 gene expression, which may indirectly lead to reduced sperm production and impaired reproductive performance in adulthood by affecting the Sertoli cell function and extracellular matrix dynamics, suggesting that abnormal testicular development in IUGR pigs may originate from altered gene expression [15]. Although recent studies have revealed the regulatory mechanisms of non-coding RNAs in the skeletal muscle and placenta of IUGR pigs [23,24], the testis-specific miRNA-mRNA network remains uncharacterized. Moreover, previous research has shown that altered gene expression in the testes of IUGR pigs can lead to reduced male reproductive capacity. Therefore, the mechanisms by which the miRNA-mRNA regulatory network affects male reproductive function remain to be elucidated.

## 2. Materials and Methods

### 2.1. Experimental Animals and Sample Collection

IUGR piglets were defined as those with birth weights below two standard deviations of the normal birth weight [1]. In this study, twelve paternal half-sibling male Duroc × Landrace × Yorkshire (DLY) crossbred piglets were selected as experimental subjects. All piglets were one day old at the time of selection. Based on birth weight, the twelve piglets were divided into two groups: six normal birth weight (NBW) piglets (mean birth weight: 1.49 ± 0.03 kg, *n* = 6) and six IUGR piglets (mean birth weight: 1.03 ± 0.05 kg, *n* = 6). All pigs were raised under standard commercial practices, and piglet castration was performed following standard commercial procedures. The operator quickly held the piglet upside down, disinfected the scrotal skin, and made a small incision in the scrotum below the testes. The testes were then extruded, and the spermatic cords were severed by scraping to achieve hemostasis, followed by removal of the testes. Hemostasis and disinfection were checked postoperatively. Subsequently, the medial parenchymal region of the testicular tissue was collected, weighed, and recorded, then rapidly frozen in liquid nitrogen and stored at −80 °C.

### 2.2. RNA Extraction and Sequencing

Three normal piglets (birth weight: 1.53 ± 0.03 kg) and three IUGR piglets (birth weight: 1.05 ± 0.05 kg) were randomly selected from the twelve pigs described above for RNA sequencing. Total RNA was extracted from the testes using TRIZOL reagent (Invitrogen, Guangzhou, China). For mRNA sequencing, library construction and sequencing were performed according to our previously described protocol [25]. Briefly, poly(A)-tailed mRNAs were enriched using Oligo(dT) magnetic beads, followed by RNA fragmentation, reverse transcription, cDNA purification, selection, re-amplification, and purification to construct the final library. Libraries that passed quality control were then sequenced to obtain genomic data. For miRNA sequencing, library construction and sequencing were conducted based on our previously reported protocol [23,26]. Briefly, RNA modifications interfering with miRNA sequencing were removed, cDNA was synthesized and amplified, and amplified fragments were recovered and purified from PAGE gels. The libraries were quantified using an Agilent 2100 Bioanalyzer (Agilent Technologies, Santa Clara, CA, USA), denatured, and diluted, followed by sequencing on the Illumina NovaSeq 6000 system (Illumina, San Diego, CA, USA). The mRNA and miRNA sequencing data were stored at the National Genomics Data Center (NGDC) (Accession numbers: PRJCA044011 and PRJCA044102).

### 2.3. Transcriptomic Data Analysis

Raw sequence data in FASTQ format were further analyzed. Quality control was performed using TrimGalore (v0.6.8, https://github.com/FelixKrueger/TrimGalore, accessed on 13 August 2024). For RNA-seq, processed data were aligned to the porcine reference genome (Sscrofa11.1, Ensembl) via Hisat2, followed by quantification with Kallisto and normalization using TPM (Transcripts per kilobase million). miRNA analysis was conducted according to our previously reported method [23,27]. Briefly, clean reads were obtained by removing adaptors and low-quality reads, and then aligned to the mature miRNA sequences from the miRBase for quantification using miRDeep2. Differential gene analysis was conducted using edgeR, with genes/miRNAs meeting *p* < 0.05 (FDR correction *p*-value) and |log2(fold change)|  > 1 defined as significantly differentially expressed genes (DEGs) or miRNAs.

### 2.4. Enrichment Analysis, Target Gene Prediction, and miRNA-mRNA Regulatory Network Construction

GO and KEGG enrichment analyses of differentially expressed genes were performed using an online platform (https://www.bioinformatics.com.cn/, accessed on 10 August 2024), and heatmaps and network diagrams were generated for visualization. Bubble plots and Sankey diagrams were generated via the OmicShare platform (https://www.omicshare.com/, accessed on 2 October 2024). Gene Set Enrichment Analysis (GSEA) and PCA plots were visualized using the OmicStudio tool (https://www.omicstudio.cn/tool, accessed on 28 November 2024). miRNA target genes were predicted using TargetScan8.0, miRDB, and miRWalk (accessed on 16 December 2024). The binding sites between miRNAs and target genes were predicted via RNAhybrid [28] (https://bibiserv.cebitec.uni-bielefeld.de/rnahybrid, accessed on 11 January 2025). Network analysis and visualization were conducted using Cytoscape (v3.9.1, http://www.cytoscape.org/, accessed on 18 February 2025) [7], network topological features including node degree and betweenness centrality were calculated using the NetworkAnalyzer plugin, and node importance was evaluated based on degree centrality using Cytoscape. Nodes with higher degree values were considered more critical in the network.

### 2.5. RT-qPCR

Quantification of miRNA and mRNA was performed as previously described in our study [29]. Briefly, RT-qPCR was conducted using TB Green Premix Ex Taq II (Takara, Kusatsu, Shiga, Japan, Cat. #RR820A) on a Bio-Rad CFX96 Real-Time PCR Detection System (Bio-Rad, Richmond, CA, USA). U6 and ACTB were used as internal controls for miRNA and mRNA, respectively, to normalize the results. Relative expression levels were calculated using the 2^−ΔΔct^ method.

### 2.6. Statistical Analysis

Data were organized using Microsoft Office Excel 2021. All results are expressed as mean ± SEM. Statistical comparisons between two groups were performed using an unpaired two-tailed Student’s *t*-test. All statistical analyses were conducted using GraphPad Prism version 9.0 (GraphPad Software, San Diego, CA, USA). Differences were considered statistically significant at *p* < 0.05 and highly statistically significant at *p* < 0.01.

## 3. Results

### 3.1. Phenotypic Differences in Testes Between NBW and IUGR Piglets

**Figure 1:**
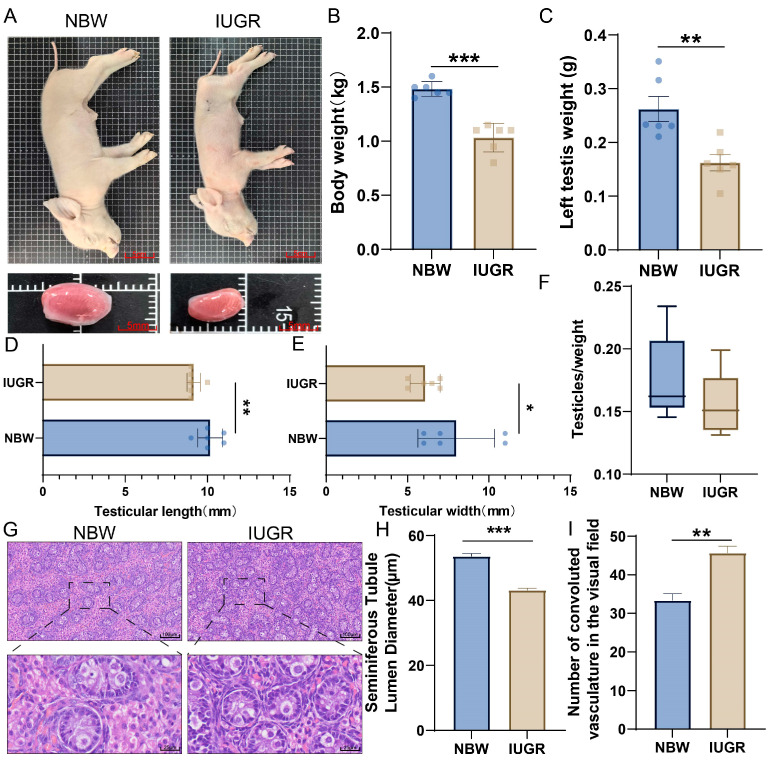
Morphological characteristics of testes in IUGR piglets. (**A**) Phenotypic characteristics of NBW and IUGR piglets. (**B**) Body weight of NBW and IUGR piglets. (**C**) Unilateral testes weight of NBW and IUGR pigs. (**D**) Testes length of NBW and IUGR piglets. (**E**) Testes width of NBW and IUGR piglets. (**F**) Gonadosomatic index of NBW and IUGR piglets. (**G**) HE-stained sections of testes from NBW and IUGR pigs. (**H**) Seminiferous tubule lumen diameter in NBW and IUGR pigs. (**I**) Seminiferous tubule density per field of view. (* *p* < 0.05, ** *p* < 0.01, *** *p* < 0.001)

The phenotypic results showed that compared to the NBW group, IUGR piglets had a significantly smaller body size and significantly smaller testes (Figure 1A). The body weight of IUGR piglets (mean: 1.03 ± 0.05 kg, *n* = 6) was significantly lower than that of the NBW group (mean: 1.49 ± 0.03 kg, *n* = 6) (Figure 1B), Additionally, the unilateral testicular weight of IUGR piglets (mean: 0.16 ± 0.02 g, *n* = 6) was significantly lower than that of the NBW group (mean: 0.26 ± 0.02 g, *n* = 6) (Figure 1C). The length and width of the unilateral testes in IUGR pigs were also significantly lower than those in the NBW group (Figure 1D,E). Compared to the NBW group, the gonadosomatic index of the IUGR group was also reduced (Figure 1F). Due to the young age of the piglets, HE-stained testicular sections showed that some seminiferous tubules were irregular in shape (Figure 1G). Compared to the NBW group, the seminiferous tubule lumen diameter in IUGR pigs was significantly decreased (Figure 1H). Due to the significant reduction in seminiferous tubule lumen diameter in IUGR pigs, the density of seminiferous tubules within the same field of view was thus significantly increased (Figure 1I).

### 3.2. Enrichment Analysis of the Top 500 Genes in the Testicular Transcriptome of NBW Piglets

**Figure 2:**
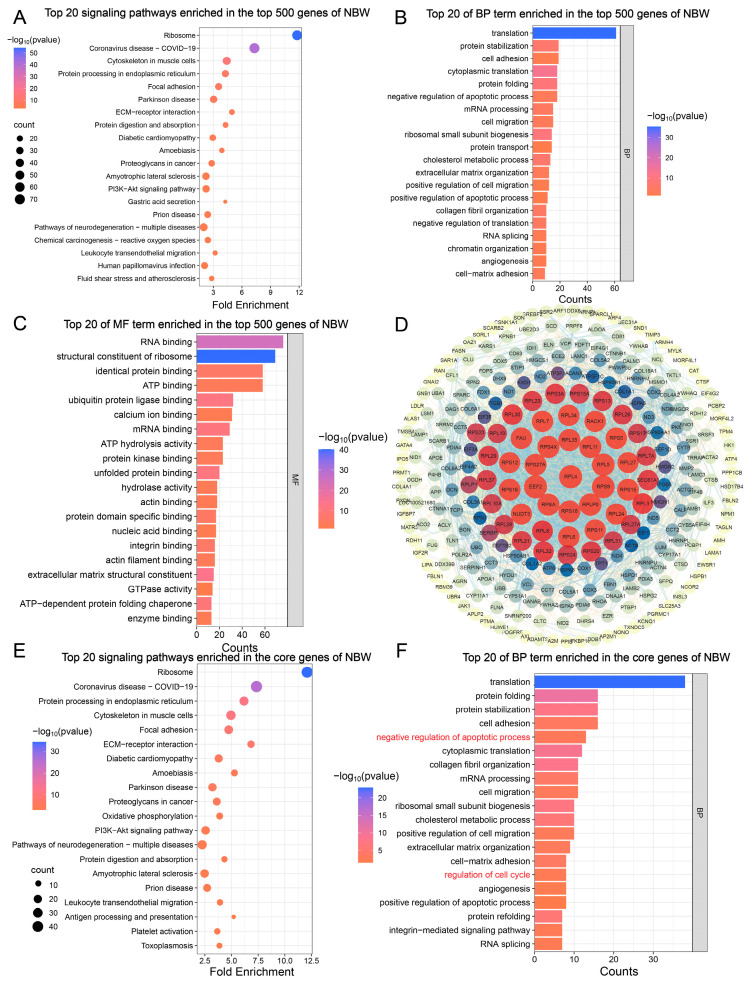
Analysis of the top 500 genes in testes of NBW pigs. (**A**) Top 20 signaling pathways of the top 500 genes in NBW piglets. (**B**) Top 20 BP of the top 500 genes in NBW piglets. (**C**) Top 20 MF of the top 500 genes in NBW piglets. (**D**) Regulatory network of the top 500 genes in NBW piglets. (**E**) Top 20 signaling pathways of core genes in NBW piglets. (**F**) Top 20 BP of core genes in NBW piglets.

We analyzed the top 500 most highly expressed genes in the testes of NBW piglets. These genes were primarily enriched in signaling pathways associated with immune-related, neurodegenerative, cardiovascular, and metabolic diseases (Figure 2A). Furthermore, they participated in key biological processes, including apoptosis and cell cycle regulation (Figure 2B, Table A1). In parallel, we investigated the molecular functions enriched among these top 500 genes, revealing that they were primarily involved in nucleic acid binding and translation regulation, protein interaction and regulation, as well as nucleotide binding and hydrolase activity (Figure 2C, Table A2). To further identify core genes within the NBW group, we constructed a protein–protein interaction (PPI) network using the STRING database. After removing unconnected nodes from the network, the resulting PPI network consisted of 280 nodes and 2029 edges, suggesting that 280 out of the top 500 genes were considered core genes (Figure 2D). Subsequent GO and KEGG analyses of these core genes indicated that they remained enriched in pathways related to neurodegenerative diseases and immune regulation. Notably, biological process enrichment revealed that these genes were involved in regulating the cell cycle and exhibited dual roles in both promoting and inhibiting apoptosis (Figure 2E,F). These findings suggest that the highly expressed core genes in NBW piglets contribute to the modulation of apoptosis and cell cycle processes.

### 3.3. Enrichment Analysis of Top 500 Genes in the Testicular Transcriptome of IUGR Piglets

**Figure 3:**
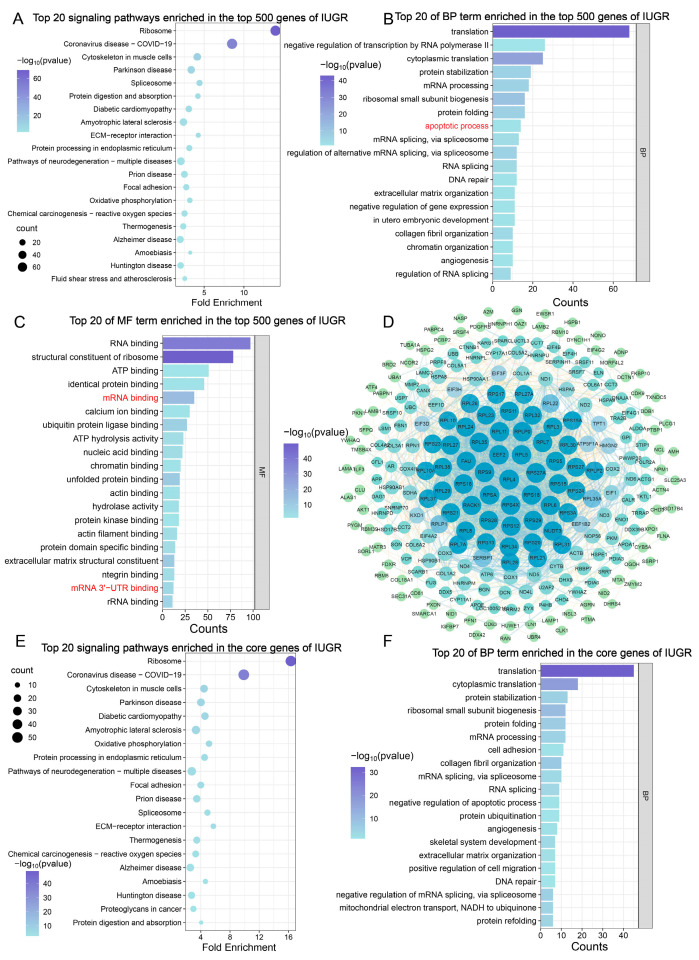
Analysis of the top 500 genes in IUGR piglet testes. (**A**) Top 20 signaling pathways of top 500 genes in IUGR piglets. (**B**) Top 20 BP of top 500 genes in IUGR piglets. (**C**) Top 20 MF of top 500 genes in IUGR piglets. (**D**) Regulatory network of top 500 genes in IUGR piglets. (**E**) Top 20 signaling pathways of core genes in IUGR piglets. (**F**) Top 20 BP of core genes in IUGR piglets.

Following our analysis of the top 500 most highly expressed genes in the testes of NBW piglets, we conducted enrichment analysis of the corresponding top 500 genes in the testes of IUGR piglets. The results showed that these genes were associated with cellular processes and structural components, and were highly enriched in neurodegenerative diseases (Figure 3A). BP enrichment analysis revealed significant enrichment of these genes in apoptotic pathways, suggesting a potential pro-apoptotic role that could disrupt normal cell proliferation (Figure 3B). MF enrichment analysis indicated that these genes exhibited RNA-binding functions, including mRNA binding (3′-UTR/5′-UTR), miRNA-mediated silencing, and translational regulation, thereby forming a tightly regulated gene expression network (Figure 3C, Table A3). This indicates that the highly expressed genes in IUGR can facilitate the establishment of a miRNA-mRNA regulatory network in IUGR piglet testes. To further identify core genes in IUGR piglet testes, we also constructed a PPI network based on highly expressed genes in IUGR. The results showed that after removing disconnected nodes, the network contained 253 nodes and 2334 edges, indicating that 253 out of the top 500 genes were core genes (Figure 3D). GO and KEGG enrichment analyses of the 253 core genes showed that they were primarily enriched in signaling pathways associated with neurodegenerative diseases, metabolic response, and stress response, which may highlight potential neuroprotective and metabolic intervention strategies to support normal piglet development (Figure 3E). BP enrichment results showed that the highly expressed core genes in IUGR piglet testes affected the occurrence of apoptosis and also influenced cell cycle progression (Figure 3F, Table A4). These results indicate that the core genes highly expressed in IUGR pigs play critical roles in modulating apoptosis and cell cycle dynamics.

### 3.4. Analysis of mRNA Differences in Testes Between NBW and IUGR Piglets

**Figure 4:**
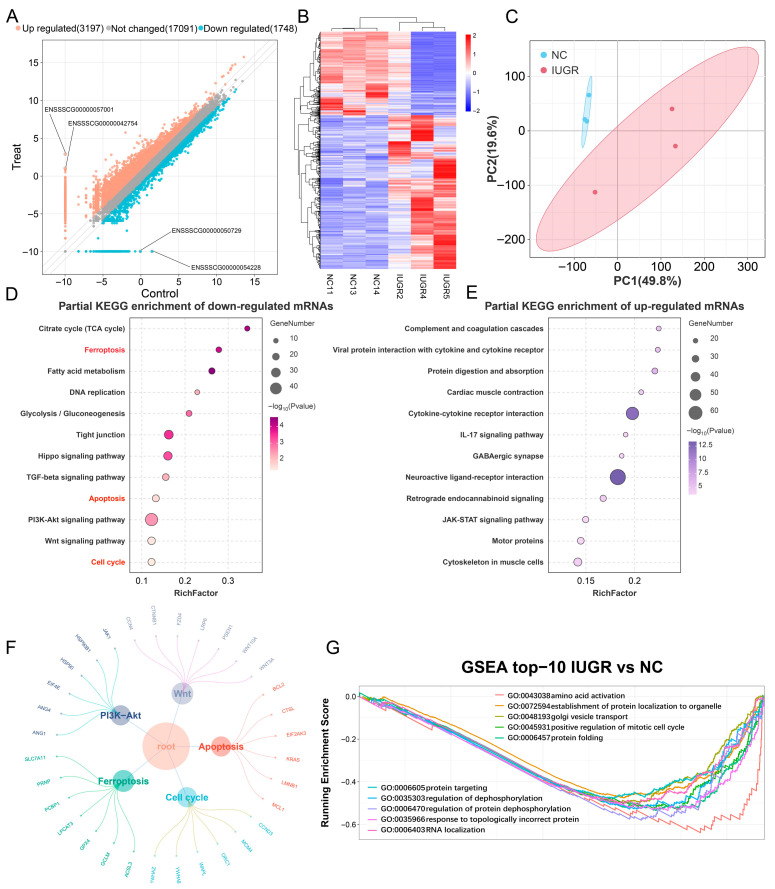
Cell cycle abnormalities in the testes of IUGR pigs. (**A**) Scatter fold change plot of differentially expressed genes in the testes of NBW and IUGR pigs. (**B**) Heatmap showing intergroup clustering patterns and differential expression profiles. (**C**) Principal component analysis (PCA) demonstrating intra- and inter-group clustering. (**D**) KEGG enrichment of down-DEGs in selected signaling pathways. (**E**) KEGG enrichment of up-DEGs in selected signaling pathways. (**F**) Signature genes of selected pathways enriched by down-DEGs. (**G**) GSEA of the top 10 BP.

After conducting enrichment analysis on the top highly expressed genes in NBW and IUGR, respectively, we further analyzed the differentially expressed genes (DEGs) in the testes of NBW and IUGR piglets. High-throughput sequencing results revealed that, compared with the NBW group, 3197 genes were significantly upregulated, and 1748 genes were significantly downregulated in the testicular tissues of IUGR piglets (Figure 4A). Hierarchical clustering heatmap analysis revealed the expression and distribution patterns of differentially expressed genes across samples, indicating clear separation between the NBW and IUGR groups into two distinct branches with good intra-group reproducibility (Figure 4B). Principal component analysis (PCA) further demonstrated distinct separation between NBW and IUGR testicular groups, with samples within each group clustering together (Figure 4C). KEGG enrichment analysis was performed separately on the upregulated DEGs (up-DEGs) and downregulated DEGs (down-DEGs). The enrichment results demonstrated that down-DEGs were primarily involved in metabolic pathways and cell death-related signaling pathways, including the TCA cycle, ferroptosis, fatty acid metabolism, DNA replication, glycolysis/gluconeogenesis, apoptosis, and cell cycle (Figure 4D). Conversely, up-DEGs were mainly associated with immune and inflammatory responses, neural signaling, and muscle system-related pathways (Figure 4E). Among the down-DEGs in the testes of IUGR pigs, marker genes associated with cell death-related signaling pathways are highlighted (Figure 4F), including key genes such as SLC7A11, GPX4, CCND3, BCL2, CTNNB1, and ANG1. Gene set enrichment analysis (GSEA) revealed that the positive regulation of the mitotic cell cycle process was significantly downregulated in the IUGR group, ranking among the top 10 enriched pathways (Figure 4G). These findings suggest that the reduced testicular size observed in IUGR pigs may result from cell cycle arrest induced by downregulated DEGs, thereby contributing to abnormal testicular development.

### 3.5. Enrichment Analysis of Top 10 miRNAs in Testes of NBW and IUGR Piglets

**Figure 5:**
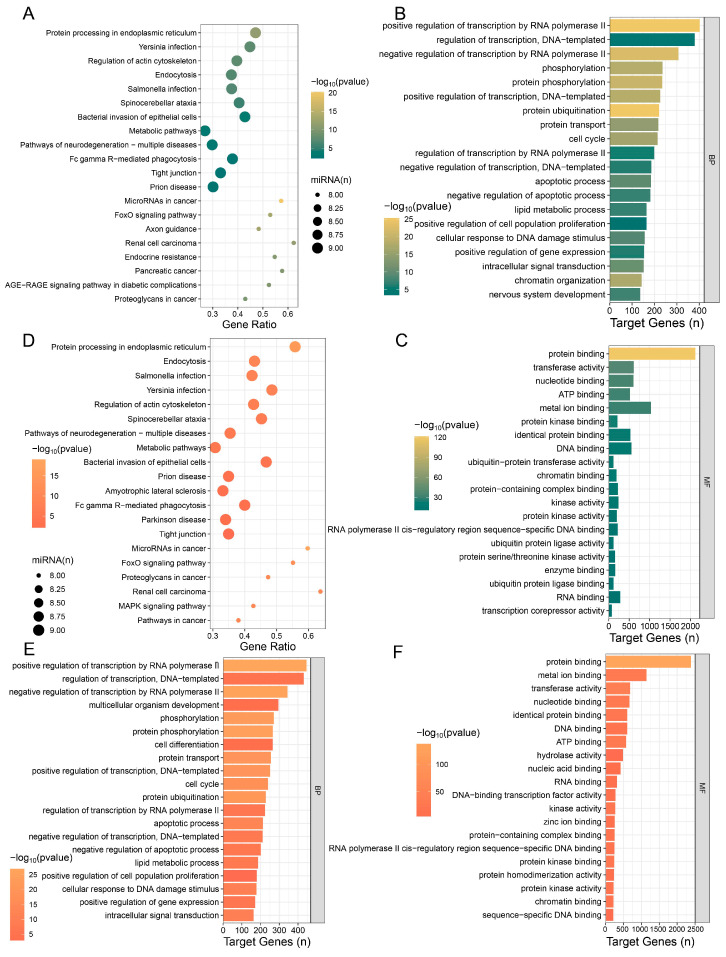
Enrichment analysis of top 10 miRNAs in testes of NBW and IUGR piglets. (**A**) Top 20 signaling pathways enriched by target genes of NBW top 10 miRNAs. (**B**) Top 20 BP enriched by target genes of NBW top 10 miRNAs. (**C**) Top 20 MF enriched by target genes of NBW top 10 miRNAs. (**D**) Top 20 signaling pathways enriched by target genes of IUGR top 10 miRNAs. (**E**) Top 20 BP enriched by target genes of IUGR top 10 miRNAs. (**F**) Top 20 MF enriched by target genes of IUGR top 10 miRNAs.

Following transcriptome analysis of NBW and IUGR piglets, we performed enrichment analyses on the target genes of the top 10 most highly expressed miRNAs in each group. The results showed that target genes of the top 10 miRNAs highly expressed in NBW were primarily enriched in fundamental cellular processes, infection and immune responses, neural regulation, and neurodegenerative pathways (Figure 5A). BP enrichment analysis revealed that these target genes were mainly enriched in biological processes including transcriptional regulation, post-translational modification and transport of proteins, cell cycle and proliferation regulation, and apoptosis (Figure 5B). Concurrently, MF analysis indicated these miRNA target genes predominantly emphasized nucleic acid interactions (Figure 5C). These findings suggest the top 10 miRNAs in NBW piglets may influence transcriptional dysregulation and protein homeostasis imbalance. We then analyzed target genes of the Top 10 miRNAs highly expressed in IUGR. KEGG enrichment analysis showed that these target genes were enriched in signaling pathways involved in maintaining cellular homeostasis, fundamental cellular structure and function, neurodegenerative diseases, and cancer development (Figure 5D). BP enrichment analysis showed primary enrichment in gene expression regulation, cell cycle, and apoptotic processes (Figure 5E), while MF analysis indicated functions in regulating nucleic acid interactions, protein interactions, and complex assembly (Figure 5F). The integrated KEGG and BP analyses suggest that the top 10 miRNAs in IUGR may modulate the cell cycle and apoptosis by regulating protein interactions and complex assembly, ultimately leading to abnormal cell proliferation and death.

### 3.6. Analysis of miRNA Differences in Testes Between NBW and IUGR Piglets

**Figure 6:**
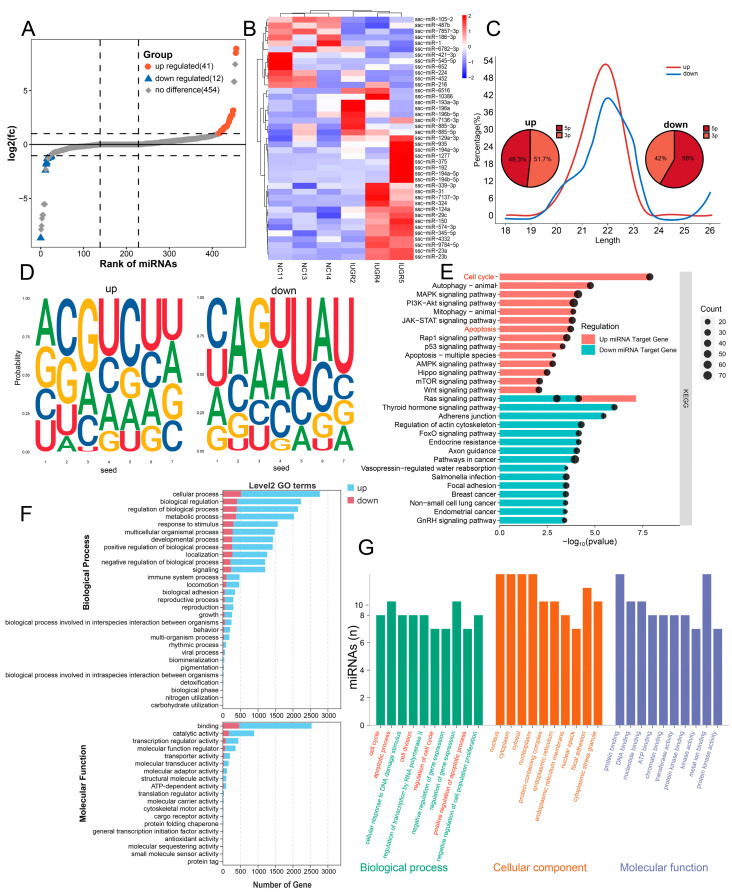
Up-miRNAs exhibit greater biological significance in IUGR testicular development. (**A**) Rank plot of differentially expressed miRNAs in NBW and IUGR piglets. (**B**) Heatmap displaying expression profiles of differentially expressed miRNAs. (**C**) Type distribution and characteristics of up-miRNAs and down-miRNAs. (**D**) Seed sequence characteristics of differentially expressed miRNAs. (**E**) KEGG enrichment analysis of target genes of up-miRNAs and down-miRNAs. (**F**) Analysis of level 2 GO terms for target genes of up-miRNAs and down-miRNAs. (**G**) GO enrichment analysis of target genes of up-miRNAs.

A total of 41 miRNAs were upregulated and 12 miRNAs were downregulated in the testes of IUGR pigs (Figure 6A). Heatmap analysis based on differentially expressed miRNAs revealed a clear separation between the NBW and IUGR groups into two independent clusters, showing high intra-group consistency (Figure 6B). Most up-miRNAs (upregulated miRNAs) were 20–22 nt in length and equally distributed between the 5p and 3p strands, whereas most down-miRNAs (downregulated miRNAs) were 20–23 nt in length and predominantly of the 5p type (Figure 6C). up-miRNAs and down-miRNAs exhibited distinct seed sequence characteristics (Figure 6D). KEGG enrichment analysis of target genes for up-miRNAs and down-miRNAs revealed that up-miRNA target genes were primarily enriched in pathways related to cell cycle and apoptosis regulation, stress response and autophagy, as well as developmental and cell polarity regulation. In contrast, down-miRNA target genes were mainly enriched in signaling pathways associated with metabolic disease regulation, neural regeneration, and infection-related mechanisms (Figure 6E). GO level 2 classification analysis of target genes for both up-miRNAs and down-miRNAs demonstrated that their target genes were broadly involved in biological processes such as cellular processes, regulation of biological processes, metabolic processes, multicellular organismal processes, developmental processes, and biological regulation (Figure 6F). Because a greater number of up-miRNA target genes in IUGR piglet testes were associated with cellular processes, biological regulation, metabolism, and development, we performed further GO analysis focused on up-miRNAs. These results showed that the target genes of up-miRNAs were significantly enriched in biological processes including the cell cycle, regulation of the cell cycle, and the positive regulation of apoptotic processes (Figure 6F,G). Previous findings revealed that down-DEGs were enriched in cell cycle processes, particularly in the downregulated positive regulation of mitotic cell cycle processes (Figure 4D,G). Combined with miRNA analysis results, these findings suggest that up-miRNAs may play a more prominent role in the biological processes underlying testicular development in IUGR.

### 3.7. Differential microRNA-mRNA Regulatory Network in Testicular Tissues Between NBW and IUGR Piglets

**Figure 7:**
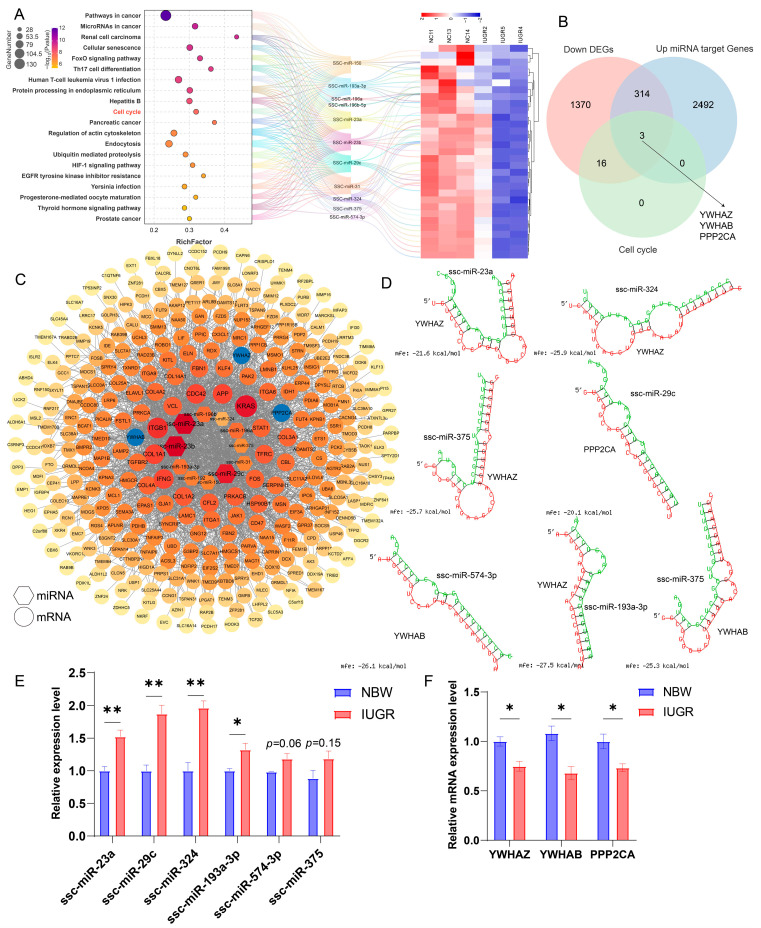
Analysis of the differentially expressed miRNA-mRNA regulatory network in the testes of IUGR pigs. (**A**) KEGG enrichment analysis of target genes of core up-miRNAs. (**B**) Venn diagram of down-DEGs, target genes of up-miRNAs, and cell cycle-related marker genes. (**C**) Network node diagram of core miRNA/mRNA interactions. (**D**) Secondary structures of ssc-miR-23a, ssc-miR-324, ssc-miR-574-3p, ssc-miR-375, ssc-miR-193a-3p, and ssc-miR-29c, with diagrams of their potential binding sites to YWHAZ, YWHAB, and PPP2CA. (**E**) RT-qPCR validation of core upregulated miRNAs. (**F**) RT-qPCR validation of target genes. (* *p* < 0.05, ** *p* < 0.01)

To investigate the relationship between differentially expressed miRNAs and mRNAs in the testes of NBW and IUGR piglets, we constructed a co-expression network of miRNA-mRNA pairs in testicular tissues. Previous results suggested that down-DEGs might contribute to abnormal testicular development in IUGR by causing cell cycle arrest, while up-miRNAs were more involved in the biological processes of IUGR testicular development. Therefore, we focused on the relationship between down-DEGs associated with cell cycle and apoptosis and up-miRNAs. We established a miRNA-mRNA regulatory network involving up-miRNAs and cell cycle-related down-DEGs. Over one-fourth of the up-miRNAs exhibited overlapping target genes with cell cycle-related downregulated differentially expressed genes, and the results demonstrated that these up-miRNAs indeed participate in cell cycle regulation. Additionally, low-expressed mRNAs in IUGR are more susceptible to regulation by these up-miRNAs (Figure 7A). Further intersection analysis of down-DEGs, target genes of up-miRNAs, and cell cycle-related markers identified YWHAZ, YWHAB, and PPP2CA as overlapping genes (Figure 7B). Co-expression network node analysis of these up-miRNAs and overlapping genes between down-DEGs and up-miRNA target genes revealed that YWHAZ, YWHAB, and PPP2CA were more likely to be influenced by miRNAs such as ssc-miR-23a, ssc-miR-23b, ssc-miR-29c, and ssc-miR-196a (Figure 7C). Further analysis revealed that YWHAZ, YWHAB, and PPP2CA may have potential binding sites with upregulated miRNAs, including ssc-miR-23a, ssc-miR-324, ssc-miR-574-3p, ssc-miR-375, ssc-miR-193a-3p, and ssc-miR-29c, with predicted binding free energies below −20 kcal/mol (Figure 7D). After identifying the core upregulated miRNAs with potential binding sites to target genes, RT-qPCR validation was performed. Consistent with the sequencing results, ssc-miR-23a, ssc-miR-29c, ssc-miR-324, and ssc-miR-193a-3p were significantly upregulated in the testes of IUGR pigs, while ssc-miR-574-3p and ssc-miR-375 showed an upward trend. The target genes YWHAZ, YWHAB, and PPP2CA were significantly downregulated in the testes of IUGR pigs (Figure 7E,F). These results suggest that the downregulated genes YWHAZ, YWHAB, and PPP2CA may have strong binding interactions with ssc-miR-23a, ssc-miR-29c, ssc-miR-324, and ssc-miR-193a-3p, warranting further investigation.

## 4. Discussion

The underlying mechanisms of IUGR remain incompletely understood, yet IUGR is recognized as a significant factor affecting livestock industry development [30]. Swine, as polytocous mammals, exhibit a notably higher incidence of IUGR piglets. Studies report that varying birth weights influence testicular development and spermatogenesis in boars [31], with male offspring of lower birth weight showing significantly reduced fertility during subsequent development [32]. Consequently, increased IUGR prevalence adversely impacts boar reproductive performance. The early identification of IUGR-affected boars with compromised fertility could mitigate such reproductive declines. The testes play a pivotal role in maintaining normal male reproductive function [33], with their development regulated by multiple cellular signaling pathways and endocrine factors [34,35]. Investigating the miRNA-mRNA regulatory networks and key miRNAs in IUGR porcine testes will advance our understanding of testicular developmental abnormalities, elucidate mechanisms underlying testicular development in piglets, and enhance their postnatal reproductive potential.

This study found that compared to NBW pigs, IUGR pigs exhibited reduced testicular volume, decreased weight, and a significantly shorter lumen diameter of seminiferous tubules, indicating testicular hypoplasia in IUGR piglets. Previous studies demonstrated that the lumen diameter of seminiferous tubules in IUGR pigs was no longer affected by birth weight at 10 months of age [18], suggesting that the impact of intrauterine growth restriction on the male reproductive system might partially diminish with postnatal growth but cannot fully offset the developmental damage caused by IUGR. It has been reported that testicular structure undergoes significant changes during gestation [15], and porcine testicular cells proliferate markedly in late gestation [36]. Our results imply that the miRNA-mRNA regulatory network in IUGR porcine testes might impair male reproductive capacity by inhibiting cell proliferation. Thus, the core up-miRNAs identified in this study potentially affect testicular development by suppressing testicular cell proliferation during late gestation.

Based on these inferences, we performed enrichment analysis on the Top 500 genes in transcriptome data from NBW and IUGR piglets and investigated core genes. Results indicate that core genes in both NBW and IUGR groups participate in regulating cell cycle and apoptosis processes. Moreover, highly expressed genes in IUGR promote mRNA binding and miRNA binding, facilitating the formation of a fine-tuned expression control network. Subsequently, we analyzed differential expression in testes between NBW and IUGR piglets. The results revealed that, compared with NBW piglets, 3197 genes were significantly upregulated and 1748 genes were significantly downregulated in IUGR testes. KEGG and GSEA enrichment analyses demonstrated that Down-DEGs were enriched in signaling pathways including ferroptosis, cell cycle, and apoptosis. GSEA results further indicated that the positive regulation of the mitotic cell cycle process was downregulated in IUGR piglets compared with NBW. These findings provide additional evidence that the proliferation of testicular cells is impaired in IUGR piglets.

miRNAs, as key molecules involved in the regulation of gene expression, have been extensively studied and numerous IUGR-associated miRNAs have been identified. It has been reported that IUGR increases oxidative stress, thereby affecting miRNA synthesis and stability through multiple pathways [13,37]. Previous studies demonstrated that miR-34a suppresses muscle growth in the prenatal skeletal muscle of IUGR pigs, while the dysregulation of miR-133a and miR-29a contributes to impaired skeletal muscle growth, making these miRNAs potential biomarkers for prenatal skeletal muscle abnormalities in IUGR pigs [21]. Upregulated ssc-miR-339-5p may play a critical role in abnormal trophoblast cell proliferation and migration [38]. Additionally, miR-574-3p is implicated in preeclampsia and participates in IUGR pathogenesis [39]. miR-193-3p has been identified as associated with placental pathology in fetal growth restriction (FGR) [40]. miR-324-5p targets genes involved in diverse pathways, including intracellular signaling transduction coordinating testicular function, spermatogenesis, and acrosome reaction, and may impact sperm production [41]. Prior to conducting differential analysis, we performed enrichment analysis on the Top 10 miRNAs in the NBW and IUGR groups. Results indicate that highly expressed miRNAs in the NBW group may influence transcriptional dysregulation, while highly expressed miRNAs in IUGR affect biological processes such as the cell cycle by modulating protein interaction networks, ultimately leading to aberrant cellular proliferation.

In this study, ssc-miR-193a-3p, ssc-miR-574-3p, and ssc-miR-324 were screened as core up-miRNAs and may serve as signature miRNAs in IUGR piglet testes. We identified 41 upregulated miRNAs and 12 downregulated miRNAs, with most miRNAs ranging between 20 and 23 Nt in length. Target genes of up-miRNAs were enriched in pathways regulating the cell cycle, apoptosis, autophagy, and development, while down-miRNAs were predominantly enriched in metabolic diseases and neurogenesis-related signaling pathways. GO analysis of up-miRNA target genes also revealed enrichment in biological processes including cell cycle and cell cycle regulation, suggesting that up-miRNAs substantially influence spermatogenesis in IUGR piglets. This indicates that up-miRNAs hold greater research significance in the testicular developmental abnormalities of IUGR piglets. Given the high correlation between the down-DEGs and up-miRNA-mediated regulation of testicular cell cycles, we propose that an miRNA-mRNA regulatory network impacts testicular development and spermatogenesis in IUGR piglets. Although numerous studies have reported miRNA-mRNA networks and signature miRNAs in IUGR pigs—primarily concerning muscle growth [29,42], intestinal health [43,44], and metabolic regulation [45]—research on male reproductive impacts remains limited. To date, no reports exist on miRNA-mRNA regulatory networks or signature miRNAs specifically in the testes of IUGR piglets.

In our study, the miRNA-mRNA regulatory network was primarily constructed based on up-miRNAs and down-DEGs related to cell cycle and apoptosis, as these were strongly implicated in IUGR-associated testicular abnormalities. While it is acknowledged that excluding up-DEGs and down-miRNAs from the network may potentially overlook additional regulatory interactions, preliminary analyses including these categories revealed that their integration did not significantly alter the core network structure focused on cell cycle regulation. However, inclusion of up-DEGs and down-miRNAs may provide complementary insights into other biological processes affected in IUGR testes, such as metabolic pathways or stress responses. Future studies could extend the network analysis to these categories to fully elucidate the complex regulatory dynamics involved.

Further investigating the miRNA-mRNA regulatory network in IUGR testes, we identified that ssc-miR-23a, ssc-miR-29c, ssc-miR-324, and ssc-miR-193a-3 bind to proliferation marker genes YWHAZ, YWHAB, and PPP2CA with binding free energies all below −20 kcal/mol. This suggests strong binding potential between these up-miRNAs and proliferation marker genes, further confirming the existence of an miRNA-mRNA regulatory network that impacts testicular development and spermatogenesis in IUGR piglets. It has been reported that ssc-miR-23a promotes fat accumulation [46,47], ssc-miR-193a-3p serves as a biomarker in the epicardial adipose tissue of piglets during hyperglycemia [48], and miR-574-3p is upregulated in the semen of infertile males [49]. Additionally, miR-574-3p inhibits mitochondrial function, reduces ATP production in GC2 cells, accelerates sperm senescence, and decreases sperm motility [50]. Numerous studies have indicated that cell cycle arrest can lead to the downregulation of YWHAZ [51,52]. YWHAZ can bind to miR-27b-5p, and its overexpression has been shown to suppress the antiproliferative effect of miR-27b-5p [52]. YWHAZ also interacts with miR-212-5p to influence cell proliferation [53]. Additionally, many studies have demonstrated that the knockout of YWHAB results in cell cycle arrest and inhibition of cell proliferation [54,55]. YWHAZ and YWHAB, members of the 14-3-3 protein family, regulate the progression of the cell cycle by interacting with various cell cycle regulatory proteins and molecules, affecting their localization, stability, and function [56,57,58]. Conditional knockout of PPP2CA in mice has been reported to disrupt spermatogonial differentiation and cause cell cycle arrest and apoptosis [59]. Furthermore, the conditional knockout of PPP2CA in murine epidermal cells leads to significant hair loss, disrupts the normal cell cycle of epidermal cells, and impairs the hair regeneration cycle [60]. These findings collectively suggest that YWHAZ, YWHAB, and PPP2CA may influence the cell cycle by interacting with different cell cycle regulatory proteins and molecules, playing a critical role in maintaining normal cell cycle progression. Based on our analytical results and previously published studies, ssc-miR-23a, ssc-miR-29c, ssc-miR-324, ssc-miR-193a-3p, along with YWHAZ, YWHAB, and PPP2CA, are likely to be key regulatory factors in the interaction network present in the testes of IUGR pigs.

A deeper understanding of the miRNA–mRNA regulatory network in IUGR piglet testes could enable the identification of at-risk individuals prior to weaning, achieving “early detection and stratification.” After detecting piglets with high-risk miRNA signatures, they could be cohorted separately and provided with high-density protein nutrition and/or immunoenhancers to reduce early mortality and shorten the compensatory growth period. This strategy would not only improve the growth and reproductive performance of IUGR individuals in the short term but also, through precision breeding, enhance the genetic gain and economic returns of the overall herd in the long term.

Despite the valuable insights provided by this study, several limitations should be acknowledged. First, the relatively small sample size may limit the statistical power and generalizability of the findings. Second, while integrated transcriptomic analysis revealed potential key regulatory networks, functional validation of the identified miRNA–mRNA interactions was not performed. Future studies with larger sample sizes and in vitro or in vivo functional assays are needed to confirm the mechanistic roles of these candidate regulators in testicular development under IUGR conditions.

In summary, this study partially fills the research gap in the male reproduction of IUGR pigs, provides potential therapeutic targets for preventing and treating IUGR-caused male reproductive disorders, and offers a theoretical foundation for further investigations.

## 5. Conclusions

This study demonstrated that, compared with pigs of normal birth weight (NBW), intrauterine growth restriction (IUGR) significantly impairs testicular development, as indicated by reduced testicular volume, decreased weight, and shortened seminiferous tubule lumen diameter. Transcriptomic comparison between the testes of IUGR and NBW pigs revealed 4945 differentially expressed mRNAs and 53 differentially expressed miRNAs. A core miRNA-mRNA interaction network was constructed, suggesting that alterations in IUGR testes may impair male reproductive capacity by inhibiting cell proliferation. Upregulated miRNAs such as ssc-miR-23a, ssc-miR-29c, ssc-miR-193a-3p, and ssc-miR-574-3p may serve as potential candidates for IUGR testes and are predicted to target core genes including YWHAZ, YWHAB, and PPP2CA, potentially acting synergistically to suppress cell cycle progression and promote apoptosis. These findings provide a foundation for future research on male reproduction in IUGR and enhance our understanding of the regulatory mechanisms underlying IUGR-associated reproductive impairment.

## Data Availability

The original contributions presented in this study are included in the article. Further inquiries can be directed to the corresponding authors.

## Abbreviations

The following abbreviations are used in this manuscript:

IUGR	Intrauterine growth restriction
NBW	Normal birth weight
DEGs	Differentially expressed genes
PE	Preeclampsia

## Appendix A

### Appendix A.1

**Table A1:** Top 25 BP terms enriched in the top 500 genes of NBW.

Top 25 BP Terms Enriched in the Top 500 Genes of NBW			
Term	Count	*p* Value	Group
translation	61	4.31 × 10^−36^	BP
protein stabilization	19	2.70 × 10^−7^	BP
cell adhesion	19	0.00521	BP
cytoplasmic translation	18	1.83 × 10^−13^	BP
protein folding	18	1.11 × 10^−9^	BP
negative regulation of apoptotic process	18	9.25 × 10^−4^	BP
mRNA processing	15	2.79 × 10^−5^	BP
cell migration	15	0.001047	BP
ribosomal small subunit biogenesis	14	3.77 × 10^−10^	BP
protein transport	14	0.04985	BP
cholesterol metabolic process	13	1.96 × 10^−7^	BP
extracellular matrix organization	12	9.22 × 10^−4^	BP
positive regulation of cell migration	12	0.009223	BP
positive regulation of apoptotic process	11	0.036481	BP
collagen fibril organization	10	7.55 × 10^−6^	BP
negative regulation of translation	10	1.59 × 10^−5^	BP
RNA splicing	10	0.001782	BP
chromatin organization	10	0.017809	BP
angiogenesis	10	0.019896	BP
cell–matrix adhesion	9	7.17 × 10^−4^	BP
response to oxidative stress	9	8.51 × 10^−4^	BP
regulation of cell cycle	9	0.03224	BP
integrin-mediated signaling pathway	8	0.012771	BP
protein localization	8	0.030255	BP
actin filament organization	8	0.03273	BP

### Appendix A.2

**Table A2:** Top 30 MF terms enriched in the top 500 genes of NBW.

Top 30 MF Terms Enriched in the Top 500 Genes of NBW			
Term	Count	*p* Value	Group
RNA binding	76	1.12 × 10^−24^	MF
structural constituent of ribosome	69	5.88 × 10^−41^	MF
identical protein binding	58	9.80 × 10^−7^	MF
ATP binding	58	3.82 × 10^−4^	MF
ubiquitin protein ligase binding	32	1.57 × 10^−12^	MF
calcium ion binding	31	0.003062	MF
mRNA binding	29	7.93 × 10^−12^	MF
ATP hydrolysis activity	23	1.44 × 10^−4^	MF
protein kinase binding	23	3.31 × 10^−4^	MF
unfolded protein binding	20	3.83 × 10^−12^	MF
hydrolase activity	18	9.34 × 10^−4^	MF
actin binding	18	0.00163	MF
protein domain-specific binding	17	2.96 × 10^−6^	MF
nucleic acid binding	17	0.003561	MF
integrin binding	16	8.43 × 10^−7^	MF
actin filament binding	16	3.42 × 10^−4^	MF
extracellular matrix structural constituent	15	6.34 × 10^−14^	MF
GTPase activity	14	0.041937	MF
ATP-dependent protein folding chaperone	13	5.12 × 10^−11^	MF
enzyme binding	13	0.001505	MF
protease binding	12	1.53 × 10^−5^	MF
transmembrane transporter binding	12	1.23 × 10^−4^	MF
heme binding	12	0.007209	MF
oxidoreductase activity	11	0.02603	MF
extracellular matrix structural constituent conferring tensile strength	10	1.75 × 10^−7^	MF
rRNA binding	10	7.58 × 10^−7^	MF
amyloid-beta binding	10	7.30 × 10^−6^	MF
mRNA 3′-UTR binding	10	9.11 × 10^−5^	MF
translation initiation factor activity	10	9.11 × 10^−5^	MF
lipid binding	10	0.01612	MF

### Appendix A.3

**Table A3:** Top 25 MFs of IUGR piglet top 500 genes sorted by *p*-value.

Top 25 MFs of IUGR Piglet Top 500 Genes Sorted by *p*-Value			
Term	Count	*p* Value	Group
structural constituent of ribosome	78	2.65 × 10^−50^	MF
RNA binding	97	3.07 × 10^−40^	MF
mRNA binding	35	1.36 × 10^−16^	MF
extracellular matrix structural constituent	13	3.94 × 10^−11^	MF
unfolded protein binding	19	4.38 × 10^−11^	MF
ubiquitin protein ligase binding	27	5.79 × 10^−9^	MF
rRNA binding	11	6.86 × 10^−8^	MF
pre-mRNA binding	7	1.22 × 10^−7^	MF
platelet-derived growth factor binding	7	1.22 × 10^−7^	MF
extracellular matrix structural constituent conferring tensile strength	10	1.91 × 10^−7^	MF
mRNA 3′-UTR binding	12	2.32 × 10^−6^	MF
NADH dehydrogenase (ubiquinone) activity	7	2.87 × 10^−6^	MF
ATP-dependent protein folding chaperone	9	3.09 × 10^−6^	MF
mRNA 5′-UTR binding	7	1.55 × 10^−5^	MF
translation initiation factor activity	11	1.60 × 10^−5^	MF
nucleic acid binding	22	2.44 × 10^−5^	MF
disordered domain-specific binding	7	2.67 × 10^−5^	MF
translation regulator activity	6	2.79 × 10^−5^	MF
ribosome binding	10	4.35 × 10^−5^	MF
amyloid-beta binding	9	6.15 × 10^−5^	MF
miRNA binding	7	6.85 × 10^−5^	MF
laminin binding	6	7.46 × 10^−5^	MF
integrin binding	13	1.09 × 10^−4^	MF
ATP hydrolysis activity	23	1.66 × 10^−4^	MF
protein domain-specific binding	14	2.34 × 10^−4^	MF

### Appendix A.4

**Table A4:** Top 30 BP terms enriched in the core genes of IUGR.

Top 30 BP Terms Enriched in the Core Genes of IUGR			
Term	Count	*p* Value	Group
translation	45	4.83 × 10^−33^	BP
cytoplasmic translation	18	3.19 × 10^−18^	BP
protein stabilization	13	2.25 × 10^−6^	BP
ribosomal small subunit biogenesis	12	5.80 × 10^−11^	BP
protein folding	12	1.30 × 10^−7^	BP
mRNA processing	12	4.64 × 10^−6^	BP
cell adhesion	11	0.021248	BP
collagen fibril organization	10	3.10 × 10^−8^	BP
mRNA splicing, via spliceosome	10	8.14 × 10^−5^	BP
RNA splicing	9	9.35 × 10^−5^	BP
negative regulation of apoptotic process	9	0.033283	BP
protein ubiquitination	9	0.047604	BP
angiogenesis	8	0.005006	BP
skeletal system development	7	2.23 × 10^−4^	BP
extracellular matrix organization	7	0.0107	BP
positive regulation of cell migration	7	0.039137	BP
DNA repair	7	0.041843	BP
negative regulation of mRNA splicing, via spliceosome	6	2.00 × 10^−6^	BP
mitochondrial electron transport, NADH to ubiquinone	6	1.58 × 10^−5^	BP
protein refolding	6	1.94 × 10^−5^	BP
regulation of RNA splicing	6	2.94 × 10^−4^	BP
regulation of alternative mRNA splicing, via spliceosome	6	7.52 × 10^−4^	BP
cell–matrix adhesion	6	0.003386	BP
regulation of apoptotic process	6	0.033365	BP
chromatin remodeling	6	0.041067	BP
regulation of cell cycle	6	0.043147	BP
positive regulation of telomere maintenance via telomerase	5	1.18 × 10^−4^	BP
alternative mRNA splicing, via spliceosome	5	1.18 × 10^−4^	BP
ribosomal small subunit assembly	5	1.74 × 10^−4^	BP
ribosomal large subunit assembly	5	2.47 × 10^−4^	BP

These appendix tables can help readers better understand the main text. The biological processes listed in the main text represent only the top 20 enriched processes, while some processes relevant to our research focus may rank between the top 20 and top 30. To maintain the flow of the main text, only the top 20 processes are visualized therein; therefore, processes ranked from the top 20 to top 30 are provided in the appendix for reader reference.
